# Parasites, stress and reindeer: infection with abomasal nematodes is not associated with elevated glucocorticoid levels in hair or faeces

**DOI:** 10.1093/conphys/cow058

**Published:** 2016-12-09

**Authors:** A. M. Carlsson, G. Mastromonaco, E. Vandervalk, S. Kutz

**Affiliations:** 1 Department of Ecosystem and Public Health, Faculty of Veterinary Medicine, University of Calgary, 3280 Hospital Drive NW, Calgary, Alberta, CanadaT2N 4Z6; 2 Reproductive Physiology Unit, Toronto Zoo, 361A Old Finch Avenue, Scarborough, Ontario,CanadaM1B 5K7; 3 Canadian Cooperative Wildlife Health Centre Alberta, 3280 Hospital Drive NW, Calgary, AB T2N4Z6, Canada

**Keywords:** Glucocorticoids, nematode, *Ostertagia gruehneri*, *Rangifer tarandus*, stress, wildlife

## Abstract

Stress hormones (glucocorticoids), incorporated into hair/fur and faeces, have been proposed as biomarkers of overall health in wildlife. Although such biomarkers may be helpful for wildlife conservation and management, their use has rarely been validated. There is a paucity of studies examining the variation of stress hormones in mammals and how they relate to other health measures, such as parasitism. Parasites are ubiquitous in wildlife and can influence the fitness of individual animals and populations. Through a longitudinal experiment using captive reindeer (*Rangifer tarandus tarandus*), we tested whether animals infected with *Ostertagia gruehneri*, a gastrointestinal nematode with negative impacts on fitness of the host, had higher stress levels compared with those that had been treated to remove infection. Faecal samples were collected weekly for 12 weeks (June–September) and hair was collected at the start and end of the study; glucocorticoids were quantified using enzyme immunoassays. Contrary to what was expected, infected reindeer had similar levels of cortisol in hair and slightly lower glucocorticoid metabolites in faeces compared with uninfected reindeer. Faecal corticosterone levels were higher than faecal cortisol levels, and only corticosterone increased significantly after a handling event. These results suggest that reindeer may use a tolerance strategy to cope with gastrointestinal nematodes and raise the question as to whether moderate infection intensities with nematodes are beneficial to the host. By removing nematodes we may have altered the gut microbiota, leading to the observed elevated faecal glucocorticoid metabolite levels in the treated reindeer. These findings demonstrate the importance of considering both cortisol and corticosterone in physiological studies, as there is mounting evidence that they may have different functionalities.

## Abstract (precis)

We used captive reindeer to determine whether *Ostertagia gruehneri*, a common gastrointestinal nematode of *Rangifer*, caused increased stress as measured by hair and faecal glucocorticoids. We found that reindeer infected with an ‘average’ dose of this parasite did not have higher stress levels compared with uninfected control animals.

## Introduction

Reindeer and caribou (*Rangifer tarandus* spp.) are iconic keystone species, important for ecosystem functioning and the socio-economic cornerstone of many indigenous cultures across the North (Nuttall, 2005; Festa-Bianchet *et al*., 2011). In the last decade, *Rangifer* herds in the circumpolar Arctic have undergone substantial declines (Vors and Boyce, 2009; Festa-Bianchet *et al*., 2011; Gunn *et al*., 2011). Thus, monitoring individual and population health to enable informed proactive management and conservation is increasingly important. However, measuring indices important for population health and fitness, such as pregnancy status, age, body condition and parasite abundance (Kutz *et al*., 2013), often requires capture and restraint or culling of the animal, which is logistically challenging, expensive and, in many cases, unfeasible or undesirable. There is a need for non-invasive integrative measures of health that can be used to track and, ideally, predict trends in individual and population health.

In recent years, the concept that the physiological state of an animal may reflect the overall health status of the individual and, ultimately, the population, has increased in popularity among wildlife ecologists and conservationists (Busch and Hayward, 2009; Dantzer *et al*., 2014). It has been proposed that glucocorticoid (GC) levels may be good indicators of an animal's physiological state (Dantzer *et al*., 2014). When an animal encounters a stressor, the activation of the hypothalamic–pituitary–adrenocortical axis and the consequent short-term elevation of GCs (primarily cortisol and corticosterone) help animals to escape threatening situations by reallocating resources away from normal maintenance activities. This stress response is necessary for survival. However, if animals are exposed to stressors for long or repeated periods of time, they may experience prolonged (chronic) elevation of GC levels, which can have deleterious effects on reproduction, survival and immunocompetence (Bonier *et al*., 2009; Busch and Hayward, 2009; Dantzer *et al*., 2014). Glucocorticoid levels can be measured in blood, hair, feathers, saliva, urine and faeces. Glucocorticoid levels in faeces represent pooled quantities of metabolized GCs from the blood and are an aggregation of steroids excreted over a period reflecting the animal's gut passage time (several hours to days). Thus, there is usually a lag between observed of peaks in blood GC concentrations and faecal GC metabolite levels (Touma and Palme, 2005). Glucocorticoids in hair are assumed to be deposited from unbound cortisol in the blood while the hair is growing, potentially representing stress over several months (Sheriff *et al*., 2011), but may also be derived from local excretions from the skin (Cattet *et al*., 2014).

If GC levels are correlated with fitness-relevant traits, their quantification in faeces and hair may be a good non-invasive method for tracking population health. However, the relationship between hair GCs and faecal GC metabolites and fitness is inconsistent in wild animals (Bonier *et al*., 2009; Busch and Hayward, 2009; Macbeth *et al*., 2010; Dantzer *et al*., 2014). To make faecal and hair stress indices useful conservation tools, they must first be validated by exploring these relationships in species- and context-specific studies (Dantzer *et al*., 2014).

On the whole, there is a paucity of published wildlife studies looking at the relationship between health indices and GC levels in faeces and hair, especially for large mammals (Bonier *et al*., 2009). By definition, a parasite affects its host negatively (Zelmer, 1998), and parasite infection can, as such, be considered a ‘stressor’, which we would expect to elicit a stress response. In spite of the ubiquitous presence of parasites in wildlife (Poulin, 2014) and their demonstrated influence on, and importance for, individual and population health (Irvine, 2006; Gómez and Nichols, 2013), very few studies have examined the relationship between stress and parasitism (Goldstein *et al*., 2005; Chapman *et al*., 2006; Monello *et al*., 2010; Cizauskas *et al*., 2015), and none has looked at these relationships in *Rangifer*.

In part, the paucity of studies may be attributable to the difficulties of studying relationships between parasitism and stress in wild animal populations. Studies are often limited by small sample sizes, burdened with confounding factors and associated with technical difficulties and high expenses. Data are especially difficult to acquire in the sparsley populated Arctic and subarctic regions where *Rangifer* reside. In cases such as these, captive animals can be good model systems and provide useful information on how GC levels interact with conservation-relevant variables.

The specific pathogen-free captive reindeer herd at the University of Calgary (treated to remove helminth infection when brought to the facility and then re-infected with specific pathogens of interest) provided a unique opportunity to explore the relationship between parasitism and stress. For the past 6 years, the gastrointestinal nematode *Ostertagia gruehneri* has been naturally cycling in the population (Hoar *et al*., 2012a). *Ostertagia gruehneri* is one of the most common *Rangifer* parasites (Kutz *et al*., 2012). It is a directly transmitted nematode, where eggs passed in faeces develop into infective-stage larvae (L3) in 3–4 weeks (temperature dependent; Hoar *et al*., 2012b) and animals are infected when they accidentally ingest them while grazing. Similar to other ostertagiines, *O. gruehneri* has two life-history strategies upon ingestion of L3. The first is to develop immediately to adult parasites (normally occurs with infections early in the season). The second strategy, which tends to occur when L3 are ingested late in the summer, is to overwinter in a hypobiotic state in the mucosa of the abomasum and mature the following spring (Kutz *et al*., 2012; Hoar *et al*., 2012a). Egg output has been shown to start 6–9 months post-infection and generally peaks in mid-summer (Irvine et al., 2000; Stien *et al*., 2002b; Hoar *et al*., 2012a). Infection with *O. gruehneri* has been associated with decreased food intake (Arneberg *et al*., 1996) and reduction in body condition and fecundity (Stien *et al*., 2002a; Hughes *et al*., 2009; Steele, 2013). Models have also demonstrated that the fitness impact of this parasite is significant enough to play a role in regulating the population dynamics of Svalbard reindeer (Albon *et al*., 2002).

In order to gain a better understanding of the variation of GC levels and how they relate to fitness-relevant traits in *Rangifer*, we conducted a longitudinal experiment to test the hypothesis that reindeer infected with *O. gruehneri* have higher faecal and hair GCs compared with non-infected reindeer.

## Materials and methods

The Animal Care Committee at the University of Calgary approved all procedures prior to implementation under protocol number AC14-0059.

### Study population

We used 10 captive female reindeer housed at the Wildlife Research Station, Faculty of Veterinary Medicine, University of Calgary, Calgary, Alberta, Canada. The herd was originally sourced from local farms in Alberta, with subsequent maintenance of the herd through in-house breeding. All animals brought into the herd underwent multiple anthelminthic treatments and a strict quarantine process to ensure that no helminth parasites were introduced to the facility. Animals were experimentally infected with the parasitic nematode *O. gruehneri* between July 2007 and November 2008, as described by Hoar *et al*. (2012a). The parasite has since been allowed to cycle naturally in the population, and its presence was confirmed by faecal flotation in all animals included in the experiment on 10 June 2014, prior to the start of the experiment. The prevalence of infection was 100%, and the average parasite intensity of animals later allocated to the ‘treated’ group (see next subsection) was 25 eggs per gram of faeces (e.p.g.; range 14.5–38.5 e.p.g.), whilst that of the ‘infected’ group was 20 e.p.g. (range 11.7–34.7 e.p.g.). Reindeer were housed on fenced 5 acre pastures. All individuals were fed the same diet consisting of wild ungulate pellets and hay and had free-choice grazing. All animals were adult females (aged 2–13 years), none were pregnant or had a calf at heel during the experimental period. All animals were marked with individual ear tags and could be easily distinguished from a distance using binoculars.

### Handling and treatment

The study ran from 18 June (day 0) to 12 September 2014 (day 86). Day 0 and day 86 were the only days when the reindeer were directly handled. On the day before handling, animals were herded from pasture and confined into smaller holding pens. This allowed for more efficient processing on the day of handling. On the day of handling (experimental set-up and end), the animals were moved through an on-site chute system. Faecal and hair samples were collected as described below.

On day 0, animals were alternately allocated to treatment groups based on the order they entered the handling facility and their age. Five animals, allocated to the ‘treated’ group, were treated to remove parasites by administering a subcutaneous injection of moxidectin on the right side above the shoulder, at a blanket dose of 0.3 mg/kg with an estimated weight of 100 kg/animal. Five animals, allocated to the ‘infected’ group, were injected with saline, using the same method and volume as described above. Hair and faeces were sampled at this time as well (see below).

Following treatment, the two groups were ‘quarantined’ separately for 2 days to minimize the risk of the treated group contaminating their new parasite-free pasture; both groups were then released into their separate but adjacent pastures. The treated group was released to a pasture that had not been grazed since its construction 6 years previously and was considered parasite free. The infected group was released into a pasture that had previously been grazed (but not overgrazed) by reindeer infected with *O. gruehneri* and was considered contaminated.

### Faecal sampling

To assess whether reindeer with parasites had higher faecal GC metabolite levels compared with reindeer treated to remove parasites, faeces were sampled directly from the rectum on the two handling days (day 0 and day 86) or, if animals were witnessed to defecate prior to entering the chute, faecal samples were collected from the ground. After handling, faecal samples were collected from each individual animal every 7 days until study end, with the exception of the last sampling period, when there were 12 days between collections. Faecal samples were obtained by observing animals in the pasture until they voluntarily defecated and collecting the samples from the ground. As faecal parasite excretion and GC levels may exhibit daily rhythms (Cavigelli *et al*., 2005; Villanúa *et al*., 2006), all faecal collections were performed between 08.00 and 12.00 h. After collection, a faecal sample was immediately placed into a zip-lock bag, and the time of collection and identity of the animal were recorded. All faecal samples were then immediately placed into a cooler with icepacks. When samples had been collected from all 10 reindeer, the samples were transported to the laboratory, frozen at −20°C, and shipped frozen to the Toronto Zoo for GC analysis, no later than 6 months post-collection.

Samples for faecal parasitology were kept in the refrigerator until analysis. The intensity (in eggs per gram of faeces) of *O. gruehneri* infection was quantified using the Wisconsin double centrifugation flotation technique, using 5 g of faeces (Egwang and Slocombe, 1982). All samples were analysed within 48 h of collection.

To test whether there was an increase in faecal GC metabolite levels in response to handling, on the final day of handling (day 86) faecal samples were collected during handling as well as ~8 and 24 h post-handling. These collection times were chosen because a previous study demonstrated an excretion lag time of GC metabolites in *Rangifer* faeces of 8–24 h (Ashley *et al*., 2011).

### Faecal glucocorticoid analysis

Faecal GC analysis has been previously validated pharmacologically for reindeer by Freeman (2008) and Ashley *et al*. (2011), whereby significant increases in faecal GC levels were observed post-adrenocorticotrophic hormone (ACTH) challenge using both cortisol and corticosterone assays with similar cross-reactivities to the enzyme immunoassays (EIAs) used in the present study. The cross-reactivities of our cortisol and corticosterone assays are 100% to the parent hormone and <5 or <1% with other GCs, respectively (Young *et al*., 2001; Watson *et al*., 2013). Glucocorticoid extraction from faeces was done as recommended by Palme (2005). In brief, 0.5 g of each faecal sample was extracted in 5 ml of 80% methanol-distilled water by rotating overnight (16–18 h) at room temperature. Samples were centrifuged for 15 min at 1200***g*** and the supernatant (faecal extract) was decanted and stored in tightly capped glass vials at −20°C until analysis. For cortisol analysis, samples were run neat as follows: 150 μl of faecal extract was dried down in a fume hood at room temperature and then the dried extracts were reconstituted in the same volume (150 μl) of EIA buffer. For corticosterone analysis, samples were diluted 1:10 with EIA buffer. Glucocorticoid concentrations were quantified in duplicate using the appropriate EIA. Faecal cortisol quantification was done using the EIA protocol described by Majchrzak *et al*. (2015). Faecal corticosterone quantification was done using the EIA protocol described by Baxter-Gilbert *et al*. (2014). Serial dilutions of pooled faecal extract showed parallel displacement with the standard curves for both cortisol (*r* = 0.99, *P* < 0.001) and corticosterone (*r* = 0.96, *P* < 0.05) assays. Recovery of exogenous hormone from pooled faecal extract was 115.4 ± 4.2% (*r*^2^_4_ = 0.99, *P* < 0.0001) and 105.5 ± 1.8% (*r*^2^_4_ = 0.99, *P* < 0.0001) for cortisol and corticosterone, respectively. Intra-assay coefficients of variation were 3.6 and 5.1%, while inter-assay coefficients of variation were 14.6 and 17.2%, for cortisol and corticosterone assays, respectively. Data are presented as nanograms of hormone per gram of faeces (ng/g). Owing to the use of both a cortisol-specific and a corticosterone-specific assay for GC metabolite detection in the faecal extracts, hormone data are presented as ‘faecal cortisol’ and ‘faecal corticosterone’ concentrations as opposed to faecal GC metabolites.

### Hair sampling

On day 0, hair from the winter coat was removed by plucking by hand, using clean gloves. Hair from the newly grown spring coat was then removed using an electric shaver fitted with a close blade for surgical preparations (Oster A5, blade no. 10). Care was taken not to break the skin. We shaved a 10 cm × 10 cm area of skin from each collection site, placing all the hair into antibacterial envelopes using clean gloves (new gloves for each animal). On day 86, each collection site was re-shaved. At this time, all the hair grown in these locations since day 0 was removed and collected as described above. The new-grown hair represented the hair grown during the experimental period. This hair was used to assess whether reindeer with parasites had higher hair GC levels than reindeer treated to remove parasites. Hair samples were stored in envelopes at room temperature before being shipped for analysis 6 months after the study ended.

Studies have shown that there is variation in hair cortisol between body regions (Macbeth *et al*., 2010; Ashley *et al*., 2011). To test this, hair was collected from two locations: from the neck, just below the base of the ear on the left side of mid-line, and from the rump, 5 cm cranial to the base of the tail to the left of mid-line. Locations were selected based on their practicality for hunter-based sample collection from harvested caribou and sampling protocols used by previous studies (Ashley *et al*., 2011; Kutz *et al*., 2013; Macbeth, 2013).

### Hair cortisol analysis

All hair cortisol quantification was performed by the David Janz research group at the University of Saskatchewan, SK, Canada. Hair decontamination, preparation and steroid-extraction methods were modified from Macbeth *et al*. (2010), which were previously validated for use in caribou (Ashley *et al*., 2011). One hundred milligrams of caribou guard hair was washed three times with 4 ml methanol for 3 min per wash on a slow rotator. After washing and drying (24 h at room temperature), hair was ground for 3 min in a Retsch MM 301 Mixer Mill (Retsch, Inc., Newtown, PA, USA; 30 Hz; 10 ml stainless-steel grinding jars; single 12 mm stainless-steel grinding ball). A slow rotator was used to extract steroids from the hair shaft by immersing 25 mg powdered hair in 0.5 ml of HPLC-grade methanol (EMD Chemicals, Gibbstown, NJ, USA) for 24 h. After extraction, each sample was centrifuged, the supernatant collected and transferred to a glass test tube. Next, each sample was rinsed two times (0.5 ml of fresh methanol per rinse) and the additional supernatant collected. Pooled supernatant was dried at 38°C under nitrogen gas. Extracts were reconstituted with 0.2 ml of phosphate buffer and cortisol was quantified in triplicate 50 μl aliquots with an enzyme-linked immunoassay kit (Oxford EA-65 Cortisol EIA kit; Oxford Biomedical, Lansing, MI, USA). Performance characteristics of the EIA kit related to accuracy and precision were reported previously (Ashley *et al.,* 2011). Data are presented as picograms of cortisol per milligram of hair (pg/mg).

### Statistical analysis

All analyses were performed using the statistical program R version 3.2.0 (R Core Team, 2015). For all analyses, assumption of normality was tested using the Shapiro–Wilks test and diagnostics plot. If this assumption was not met, the appropriate adjustment was made (such as logarithmic transformation of the data) or non-parametric tests were used. Non-parametric tests were also performed when sample sizes were <30. The significance level was set at *P* < 0.05.

The effect of moxidectin treatment on *O. gruehneri* faecal egg counts was tested using a Mann–Whitney *U*-test, using all available data.

For cortisol, linear mixed-effect models, using the *lme* function from the *nlme* package, were used to test whether there was a difference in GC levels between the two treatment groups. To satisfy assumptions of normal distribution, faecal cortisol data were logarithmically transformed. Similar transformations did not normalize corticosterone data, and the function *glmer.nb* in the *lme4* package that fits a generalized linear mixed-effect model for the negative binomial family was used for analysis. For these models, treatment group (with the ‘treated’ reindeer group used as the reference level), reindeer age, day of faecal collection (sampling day) and time of faecal collection (sampling time) were used as fixed effects (predictors). Faecal cortisol levels appeared to follow an approximate 21 day cyclical pattern. The cycle appears to have three phases, a peak (phase 1), a trough (phase 2) and a midpoint (phase 3). A separate model replacing sampling day (linear predictor) with dummy variables, representing the phases of the cycles, was fitted to mimic the pattern observed: phase 1 [sampling days at the (peak) of the cycle; 7, 28, 49 and 70], phase 2 (sampling days at the trough in the cycle; 14, 35, 56 and 82), and phase 3 was modelled as the reference level. To test whether *O. gruehneri* intensity (as measured by egg counts) was a significant predictor of faecal cortisol and corticosterone levels, we ran separate *lme* and *glmer.nb* models using only the infected reindeer. To account for repeated sampling of individual animals (Paterson and Lello, 2003), animal identity was fitted as a random effect in all above described models. Model simplification was performed by step-wise deletion from a maximal model. Model comparison was done using likelihood ratio tests, where *lme* models were fitted by maximum likelihood. Faecal samples used for the analysis described above were those collected from day 7, the first day on which parasite burdens were visibly reduced in the treated group, to day 82, the last day before the final handling event. We tested whether models with an auto-correlation structure improved the fit of the models (as described by Pollitt *et al*., 2012), but they did not and an auto-correlation structure was, therefore, not included in the models presented here.

To test whether faecal cortisol and corticosterone levels were correlated, we ran a Spearman correlation test. We used a Wilcox signed rank test to examine whether there was a significant difference between faecal cortisol and corticosterone values.

To test whether there was a significant difference in hair cortisol levels between the two treatment groups, we used a Mann–Whitney *U*-test. Paired Wilcox signed ranked tests were used to examine whether there was a difference in hair cortisol levels from hair collected from the neck and rump. After logarithmically transforming data, *F*-tests were used to compare the variances of cortisol levels between the two sampling sites. We tested for differences in hair cortisol between body regions for both old-growth hair (grown in year *t* − 1 and plucked at day 0) and re-grown hair (hair grown during the study period and shaved at day 86). A Spearman's rank correlation test was used to test for a correlation between hair cortisol (from hair collected at the end of the study) and faecal cortisol and corticosterone. To do this, faecal cortisol and corticosterone were averaged across the experimental period (from day 7 to day 86) for each individual animal.

To test whether GC levels were higher in faecal samples collected 8 and/or 24 h post-handling, we used paired Wilcox signed ranked tests.

## Results

We collected a total of 149 faecal samples from 10 individual reindeer over a period of 12 weeks. Faecal samples were collected and analysed for faecal GC levels and parasites from all individuals at all time points, with two exceptions. On day 0, we were unable to collect a faecal sample from one individual during the time it took to complete handling. On day 82, one animal had not voluntarily defecated before 12.00 h and thus no sample was recovered from that individual on that day. A total of 99 faecal samples were collected between days 7 and 82.

### Moxidectin treatment

**Figure 1:**
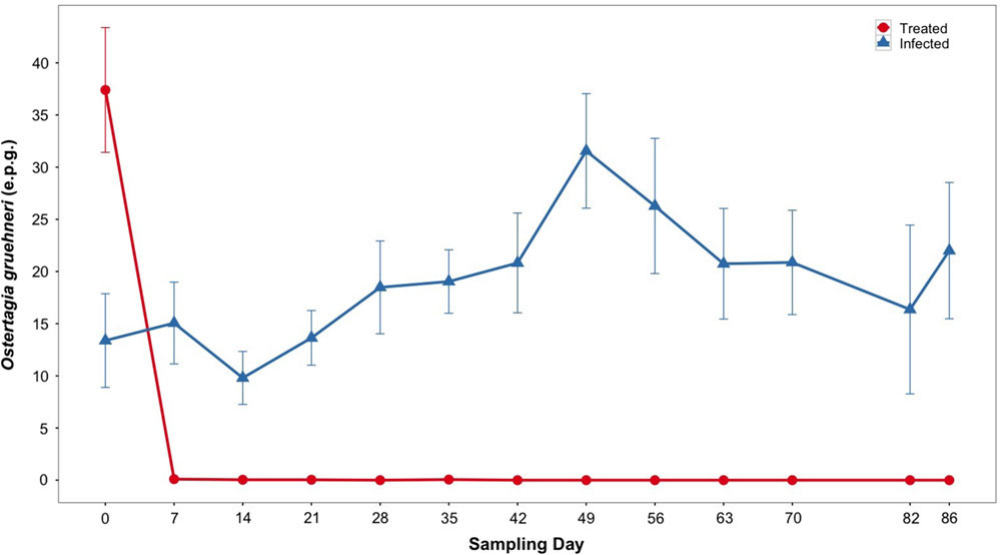
Reindeer treated with moxidectin (red circles and red line) had significantly lower parasite (*Ostertagia gruehneri*) intensity compared with infected reindeer (blue triangles and blue line) from day 7 onward. Parasite intensities (measured as eggs per gram of faeces; e.p.g.) are represented here as a mean value (±SEM) for each sampling day.

Treatment with moxidectin significantly reduced *O. gruehneri* faecal egg counts to zero on day 7 of the experiment, and counts remained thus throughout the experiment (*W* = 2472.5, *P* < 0.001; Fig. 1), with the exception that on five occasions one egg was found in a sample of ~5 g of faeces from four different animals. Faecal egg counts peaked in July in the infected group (Fig. 1).

### Faecal glucocorticoids

**Figure 2:**
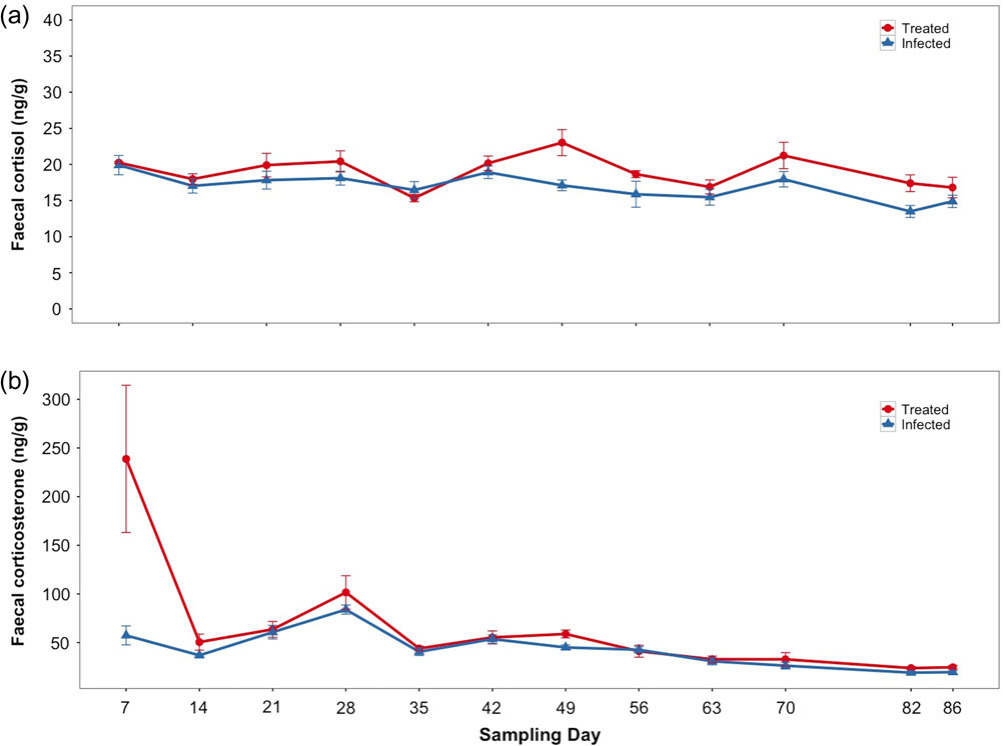
Reindeer infected with *Ostertagia gruehneri* (blue triangles and blue lines) had lower faecal cortisol (**a**) and faecal corticosterone (**b**) than treated reindeer (not infected with *O. gruehneri*; red circles and red lines). Glucocorticoid values (in nanograms per gram) are represented here as a mean value (±SEM) for each sampling day.

Infected (parasitized) reindeer did not have higher faecal cortisol or corticosterone levels compared with treated (non-parasitized) reindeer, and there was relatively little between-individual variation in faecal GC levels (Table 1). Treatment group was a significant predictor of faecal GC levels (cortisol: *P* = 0.02 and corticosterone *P* < 0.001; Table 1), but the difference in faecal GC levels was small (Fig. 2). Infected reindeer had, on average, 10% lower cortisol and 18% lower corticosterone compared with treated reindeer. Cortisol or corticosterone levels did not increase with faecal egg counts (*P* = 0.84 and *P* = 0.67, respectively; Table 1). Sampling day was a significant predictor for both GC metabolites measured (Table 1 and Fig. 2). For cortisol, the model fitting sampling day as a ~21 day cycle was a better fit than the model where sampling day was treated as a linear covariate. For corticosterone, no clear cyclical pattern was observed over time, and the model with a linear covariate was a better fit (see Fig. 2). Corticosterone levels decreased slightly over time. Sampling time or reindeer age did not explain any variation in faecal cortisol or corticosterone levels (Table 1).

**Table 1:** Summary of the minimal linear mixed-effects model and the generalized linear mixed-effects model testing the effect of parasitism on faecal cortisol and corticosterone levels

Models used	Linear mixed effect model					Generalized linear mixed effect model				
Response	Log[cortisol]					Corticosterone				
Fixed effects	Coeff.	SE	*F*-value	d.f.	*P*-value	Coeff.	SE	*z*	d.f.*	*P*-value
Intercept	**2.94**	**0.03**	**21459.13**	**87**	**<0.001**	**4.85**	**0.09**	**51.71**	**88**	**<0.001**
Treatment group	**−0.11**	**0.04**	**7.91**	**8**	**0.023**	**−0.28**	**0.09**	**−3.29**	**8**	**<0.001**
Sampling day						**−0.02**	**0.002**	**−9.42**	**88**	**<0.001**
Phase 1	**0.08**	**0.04**	**17.89**	**87**	**<0.001**					
Phase 2	**−0.09**	**0.035**	**7.49**	**87**	**0.008**					
Sampling time			1.66	86	0.20			0.54	84	0.44
Age			2.22	7	0.18			1.33	7	0.24
**Random effect**	SD 0.031					SD 9.04 × 10^−6^				

**Fixed effect**			***F*-value**	**d.f.**	***P*-value**			*F*-value	d.f.	*P*-value
*Ostertagia gruehneri*			0.0417	44	0.839			0.12	44	0.74
**Random effect**	SD 0.027					SD 4.2 × 10^−6^				

Coefficient estimates (Coeff.) and standard errors (SE) associated with *F*-values (linear mixed-effects model; lme) or Walds *z*-scores (generalized linear mixed-effects model; glmm) and significance levels are reported. Terms in the minimal model are represented in bold numbers and excluded terms in standard font. For Treatment group, the reference group is the ‘treated’ group. Separate models were run to test whether *Ostertagia gruehneri* intensity (in eggs per gram of faeces) was a significant predictor of glucocorticoid levels using data from the infected group only. To control for repeated sampling of individual animals, animal identity was fitted as a random effect in all models. *Degrees of freedom (d.f.) are not calculated for glmms; values reported for corticosterone are based on outputs from an lme model using the same data set.

**Figure 3:**
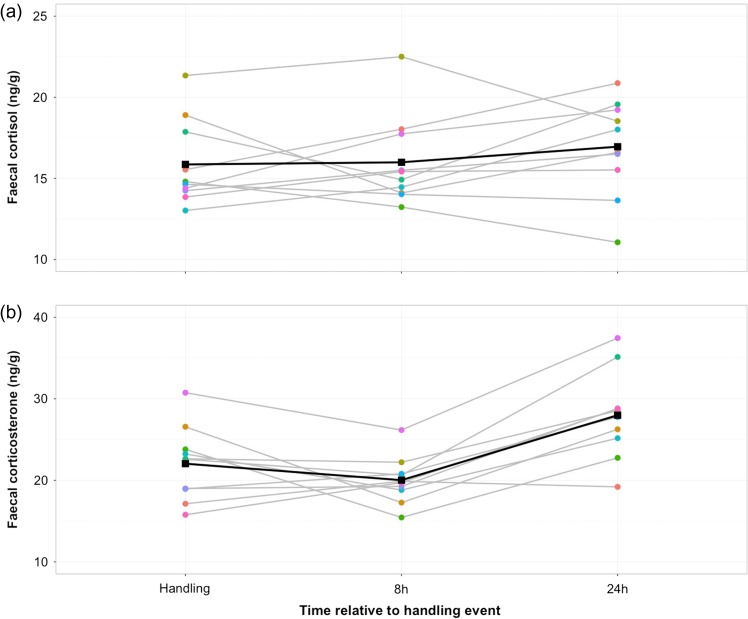
There was no significant increase in cortisol levels 8 or 24 h after handling (**a**), but there was a significant increase in corticosterone levels 24 h after handling (**b**). Faecal cortisol and corticosterone levels for individual animals are represented by different coloured circles connected by grey lines. Means are shown as black squares connected by black lines.

There was no significant increase in faecal cortisol or corticosterone levels 8 h after the reindeer were handled (*V* = 24.5, *P* = 0.79 and *V* = 40, *P* = 0.23, respectively). There was a significant increase in corticosterone levels 24 h after handling (*V* = 3, *P* = 0.01; Fig. 3b), with an average increase of 30% (±8.9). However, this pattern was not consistent between all individuals; two reindeer had a decrease in corticosterone levels 24 h after handling. Although there was a trend for higher faecal cortisol levels 24 h after handling, this was not significant (*V* = 19, *P* = 0.43; Fig. 3a).

Faecal corticosterone levels (mean, 50.87 ng/g; range, 10–446 ng/g) were significantly higher (*P* < 0.01) than faecal cortisol levels (mean, 18.80 ng/g; range, 10.59–50.64 ng/g) and had a wider range. The average cortisol/corticosterone ratio was 0.3. Faecal cortisol and faecal corticosterone levels within an individual showed a significant positive correlation (*P* < 0.01).

### Hair cortisol

**Figure 4:**
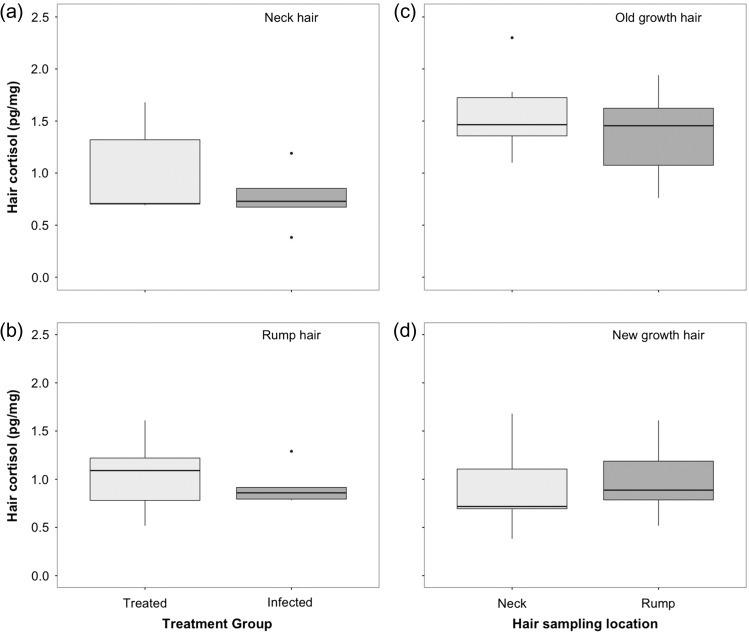
There was no significant difference in hair cortisol levels (in picograms per milligram) from hair collected from the neck (**a**) or rump (**b**) between treated (light boxes) and infected reindeer (dark boxes) or between hair collected from the neck (light boxes) or rump (dark boxes) in old-growth (**c**) or new-growth hair (**d**).

There was no significant difference in hair cortisol levels between the two treatment groups for hair collected from from the neck (*W* = 16, *P* = 0.55; Fig. 4a) or the rump (*W* = 13, *P* = 1; Fig. 4b). There was no difference in hair cortisol levels between the sampling sites for old-growth hair (*P* = 0.43; Fig. 4c) or new growth hair (*P* = 0.23, Fig. 4d) and no significant difference in variation between two groups (new-growth, *F*_9_ = 1.69, *P* = 0.44; and old growth, *F*_9_ = 0.41, *P* = 0.20). Furthermore, faecal cortisol or corticosterone levels (averaged over the study period) did not correlate with cortisol levels in either neck (*P* = 0.81 and *P* = 0.58, respectively) or rump hair (*P* = 0.97 and *P* = 0.39, respectively).

## Discussion

Reindeer infected with the parasite *O. gruehneri* did not have higher levels of glucocorticoids in faeces or hair compared with uninfected reindeer as hypothesized. Instead, the infected group had statistically significantly lower faecal cortisol and corticosterone levels compared with the treated (parasite-free) group. However, compared with other studies, the differences were small (the infected group had, on average, 10 and 18% lower faecal cortisol and corticosterone levels, respectively). For example, in an experimental manipulation, Sheriff *et al*. (2009) found that snowshoe hare dams exposed to an environmental stressor (predation) had up to 837% higher faecal GC metabolite levels. In addition, several studies have reported at least a 4-fold increase in faecal GC metabolite concentrations after pharmacological stimulation of the adrenal system (Miller *et al*., 1991; Dehnhard *et al*., 2001; Sheriff *et al*., 2009; Mastromonaco *et al*., 2014).

A lack of response to parasitic infection in faecal and hair GCs may occur if these substrates do not accurately reflect adrenal activity (Touma and Palme, 2005); this is, however, unlikely in our case. The use of cortisol and corticosterone immunoassays with similar cross-reactivities to the ones used in our study have been previously validated pharmacologically for various cervid species (cortisol, Christofoletti *et al*., 2010; corticosterone, Millspaugh *et al*., 2002). In reindeer specifically, faecal GC metabolite measurements were previously validated pharmacologically by both Freeman (2008; cortisol and corticosterone immunoassays) and Ashley *et al*. (2011; corticosterone immunoassay) using exogenous ACTH to stimulate the release of endogenous GC from the adrenal glands. Freeman (2008) and Ashley *et al*. (2011) found an increase in faecal GC metabolites 11 and 8 h after injection, respectively. Furthermore, we found a significant increase in faecal corticosterone levels after a handling event, which suggests that the substrate accurately reflects adrenal response. Trapping, confinement and handling events have been used as biological validations of faecal GC metabolite concentrations in other studies (Touma and Palme, 2005; Wilkening *et al*., 2013; Hämäläinen *et al*., 2014). Although the 30% increase in faecal corticosterone levels observed here is relatively small in comparison to responses to pharmacological stimulation of the stress axis through ACTH challenge recorded in other species (Touma and Palme 2005), it is on par with the findings of Ashley *et al*. (2011) for reindeer.

This study was limited to 10 reindeer, and small sample sizes could have limited our ability to detect a stress response in hair; however, our experimental study design and longitudinal faecal sample collection granted considerably more power to detect subtle changes. Indeed, we did detect the small difference in faecal cortisol and corticosterone levels between the two treatments as statistically different. Thus, it appears as if nematodes, within the range of infection intensities observed in this study, do not increase physiological stress levels. Our results do, however, indicate that removal of nematodes might increase GC levels, although the biological significance and implication of the small difference observed between groups is unclear.

Other studies evaluating the relationship between parasites and GCs have found varying results. Some found a positive but often context-dependent association, whereas others found no relationships at all (Fleming, 1997, 1998; Chapman *et al*., 2006; Pedersen and Greives, 2008; Monello *et al*., 2010; St Juliana *et al*., 2014). The few studies that have looked at natural infections in ungulates showed that parasite burdens are not associated with increased faecal GC metabolite levels (Goldstein *et al*., 2005; Cizauskas *et al*., 2015). In fact, our results echo findings presented by Cizauskas *et al*. (2015), in which a negative association, or lack of association, between stress hormones and macroparasites was found in zebras and springboks in Nambia. They proposed that this was attributable to hosts adopting a tolerance strategy to gastrointestinal parasites. It is generally recognized that hosts can defend against pathogens using the following three main strategies: avoidance, which reduces the risk of exposure; resistance, which reduces the pathogen burden once infected; and tolerance, which reduces the loss of fitness caused by the infection (Råberg *et al*., 2009; Medzhitov *et al*., 2012). Resistance is a function of the immune system and, although it is crucial for the host to protect itself from infection, it usually carries a substantial fitness cost (Medzhitov *et al*., 2012). Although there is a role of acquired immunity in controlling infection levels of some nematodes, the abundance of *O. gruehneri* is high in adult animals, suggesting that acquired immunity may not play a big role (Irvine *et al*., 2001). Furthermore, gastrointestinal parasites are ubiquitous in wild ungulate populations (Samuel *et al*., 2001) and, although infection with gastrointestinal nematodes has known fitness impacts, they are usually subclinical (Stien *et al*., 2002a), suggesting that virulence is low. Tolerance mechanisms, and not resistance mechanisms, are usually selected for when virulence is low (Medzhitov *et al*., 2012). Thus, it is possible that reindeer have adapted a tolerance to gastrointestinal nematodes and, therefore, do not experience infection as a stressful stimulus.

Tolerance may be dose dependent, whereby at high doses (intensities) a parasite infection may become deleterious to the host (Schneider and Ayres, 2008; Ayres and Schneider, 2012). In the present study, parasite intensities were relatively low, but within the range of what has been reported for wild caribou (Hoar *et al*., 2009; Kutz *et al*., 2012). Overall, *Rangifer* have low-intensity infections of strongyles (from very few eggs per gram of faeces up to a few hundred) compared with domesticated livestock (commonly thousands of eggs per gram of faeces; Hoberg *et al*., 2001; Hrabok *et al*., 2006; Hoar *et al*., 2009). Usually, only very high worm burdens are associated with clinical signs (Hoberg *et al*., 2001). Although lower infection intensities may have subclinical impacts on body mass and fecundity (Stien *et al*., 2002a), this may not translate into a physiological response. Indeed, in experiments using dairy calves (Fleming, 1998) and lambs (Fleming, 1997), only when animals were inoculated with high doses of infective larvae (200 000 *Ostertagia ostertagi* larvae in calves and 20 000 *Haemonchus contortus* larvae in lambs) was there an increase in circulating cortisol concentrations. The lower doses (10 000 and 100 000 larvae in calves; 0 and 2000 larvae in lambs) appeared to have no effect. Furthermore, trickle infections, where adult sheep were infected with *H. contortus* over several weeks (better mimicking a natural infection), failed to produce a cortisol response (Shutt *et al*., 1990), suggesting that perhaps only acute infections (single inoculations) with high parasite loads produce a stress response. This is in line with what we would expect if animals have adapted a tolerance strategy to cope with parasite infection.

Alternatively, moderate infection with nematodes may be beneficial to the host, and removing them may increase stress levels. Helminths, such as nematodes, have an immunoregulatory capacity that creates an anti-inflammatory environment, which can be beneficial for hosts. Indeed, the ‘hygiene hypothesis’ was proposed in the 1970s when members of a rural indigenous community with high levels of viral, bacterial and helminth infections were observed to have lower incidences of allergic disease compared with a community with low levels of infection. Today, helminths are used in human medicine to control excessive inflammatory responses, such as autoimmune disease and inflammatory bowel disease (Helmby, 2015). However, the mechanisms by which helminths regulate immune responses are not clear. It has been hypothesized that helminth-induced modifications of the gut microbial community (microbiota) may be responsible for the therapeutic properties of helminths (Giacomin *et al*., 2015). The composition and structure of the microbiota is important for host health because it provides essential host services, such as nutrient absorption, immune system development and behaviour regulation. Helminths have co-evolved with their hosts, are prevalent in livestock, humans and wildlife and are essential parts of biological communities (Lafferty *et al*., 2008) and are often an overlooked component of the gastrointestinal biota. Studies have demonstrated that helminths can interact with the gut microbiota, and infection may alter the gut microbiome, with potential downstream effects on gut function and host health (Broadhurst *et al*., 2012; Lee *et al*., 2014; Kreisinger *et al*., 2015). Furthermore, the gut microbiota forms part of a complex network with bidirectional communication, dubbed the gut–brain axis. Signals/changes on either end of the axis have the potential to influence the hypothalamic–pituitary–adrenocortical axis and, thus, GC levels (Palma *et al*., 2014; Rea *et al*., 2016). Indeed, studies have shown that germ-free animals have elevated corticosterone levels compared with animals with gastrointestinal bacteria (reviewed by Rea *et al*., 2016). Thus, it is possible that by removing parasites native to the host population, we caused a distruption in the gut microbiota leading to the observed increase in GC levels in the treated group.

Our study occurred during a period of active hair growth (86 days). Faecal cortisol and corticosterone averaged across the experimental period and hair cortisol levels from hair collected at the end of the study should, therefore, each represent systemic hypothalamic–pituitary–adrenocortical activity during the experimental period. All the same, we found no significant correlation between mean faecal cortisol or corticosterone and hair cortisol levels. Studies examining the relationship between cortisol in hair and other sample matrices, such as saliva and faeces, show conflicting results (Meyer and Novak, 2012; Bryan *et al*., 2013; Mastromonaco *et al*., 2014). The precise mechanism of GC integration into hair remains undetermined. Although passive supply from the vascular system during hair growth is believed to be the major route, there is evidence suggesting that hair cortisol may also be derived from the skin. For example, Cattet *et al*. (2014) showed that hair cortisol levels varied between capture methods and were elevated in captured animals even in the hair's quiescent (non-growing) phase (Cattet *et al*., 2014). If faecal and hair cortisol are not derived from the same source, one would not expect GC levels in these substrates to correlate. The methods used for extraction and quantification of GCs can also influence results, and here we used different laboratories and methods for hair and faecal GC analysis (Touma and Palme, 2005).

Unlike previous studies in reindeer (Ashley *et al*., 2011) and grizzly bears (Macbeth *et al*., 2010), we did not find any variation in hair cortisol between body regions despite using the same laboratory and methods for quantification of hair cortisol. Given that we analysed both ‘new-growth’ (grown during the study period) and ‘old-growth’ hair (the winter coat grown in the previous year), it is unlikely that the difference in results between our study and previous studies is attributable to a seasonal effect of sampling. It is unclear why Ashley *et al*. (2011) detected a significant difference between body regions in *Rangifer*, whereas we did not. As discussed above, discrepancies between studies may be attributable to differences in hair collection methods and variation in local secretion of cortisol from the skin. Until these issues are resolved, we recommend that hair be collected from the same site, using standardized methods, if longitudinal and between-individual comparisons are to be made.

To our knowledge, this is the first longitudinal study on *Rangifer* to present data on both faecal cortisol and corticosterone metabolites from the same samples (Freeman, 2008; Ashley *et al*., 2011; Wasser *et al*., 2011; Macbeth, 2013; Yin *et al*., 2015), and it has revealed several noteworthy patterns that would have been missed in a cross-sectional study. First, analysis revealed what appears to be a 21 day cyclic pattern in faecal cortisol levels that was not present in faecal corticosterone levels. Glucocorticoid levels may fluctuate in response to the oestrous cycle (von der Ohe and Servheen, 2002; Fanson *et al*., 2014), which is ~20 days in reindeer (Ropstad, 2000). However, our study was conducted in summer, during anoestrus. Nonetheless, there is evidence demonstrating that cervids develop ovarian follicles in a wave-like fashion even during anoestrus (Asher *et al*., 1997; McCorkell *et al*., 2004), which could be related to the cycle observed. These cycles are, however, expected to be shorter than 21 days (Asher *et al*., 1997; McCorkell *et al*., 2004). Environmental conditions (temperature, rainfall, humidity etc.) can also influence GC levels (Millspaugh *et al*., 2001; Frigerio *et al*., 2004; Weingrill *et al*., 2004). There was no clear and consistent pattern observed in faecal cortisol or corticosteone levels and temperatures 1–3 days before sampling. It is possible that the observed cycle, or cycle length, is an artefact of our sampling design. More frequent sampling and a longer time series are needed to establish firmly the presence and length of a cycle and the underlying cause.

Koren *et al*. (2012) quantified both cortisol and corticosterone in blood from the captive reindeer herd at the University of Calgary using mass spectometry and found that cortisol was the dominant steroid (cortisol:corticosterone ratio >5). However, in cervids the circulating cortisol is metabolized to corticosterone-like molecules in the faeces (Wasser *et al*., 2000). Consistent with this, we found higher concentrations of corticosterone than cortisol in faeces, with a cortisol:corticosterone ratio of 0.3. Other researchers have also shown that species that are cortisol dominant in the blood register higher corticosterone than cortisol levels in faeces (sea lion, Mashburn and Atkinson, 2004; coyote, Stevenson, 2015; cattle, Morrow *et al*., 2002). The discrepancy in faecal GC output can be attributable to various factors, including steroid metabolism in the species, extraction method and immunoassay type, which highlights the importance of choosing the appropriate methods for faecal hormone analysis (Touma and Palme, 2005).

Finally, although we found an overall positive correlation between faecal corticosterone and cortisol, there was a differential response demonstrated by these steroids. It is generally assumed that cortisol and corticosterone share the same physiological roles in free-ranging mammals, that their relative importance depends on their concentrations and that the concentration of the two steroids should be positively and strongly correlated as a result of their linked synthesis. However, we found a wider range of corticosterone than cortisol values. There were also two ‘outliers’ for corticosterone, where two individuals exhibited high corticosterone values (446.83 and 251.65 ng/g) while the corresponding cortisol values were well within the observed range (19.94 and 20.29 ng/g). These ‘outliers’ occurred on day 7, causing a clear peak in corticosterone levels but not cortisol levels in the treated group. The peak could reflect the host response to dying parasites in the mucosa and lumen of the gut or a response to changes in group dynamics and social status because of the splitting and regrouping of the original ‘herd’ (Creel *et al*., 2013). Furthermore, there was a measurable significant increase in corticosterone but not cortisol after a handling event. And finally, we observed a cyclic pattern of faecal cortisol but not corticosterone levels.

There is some evidence to suggest that cortisol and corticosterone may signal independently (Koren *et al*., 2012). For example, a switch from corticosterone dominance to cortisol dominance in response to chronic stress has been observed in rabbits (Kass *et al*., 1954), serum GCs exhibited different patterns in response to acute stressors in llamas (*Lama guanicoe*; Ovejero *et al*., 2013), and in rodents (*Ctenomys talarum*) the plasma cortisol increased after an ACTH challenge but corticosterone did not, even though corticosterone levels were higher than cortisol levels (Vera *et al*., 2012). Furthermore, rodents also had significant inter-annual variation in the levels of corticosterone, which resulted in a pronounced change in the cortisol: corticosterone ratio in plasma. Although our results provide some support to the hypothesis that cortisol and corticosterone are affected differently by environmental stressors and have different roles in physiological functions, a study specifically designed to test that theory would be needed to confirm this. Furthermore, comparisons of the responses of cortisol and corticosterone are relatively rare, and in the absence of literature with reliable measures of both GCs in the same sample it is difficult to interpret the biological implications of our results.

In summary, we found that GC levels in hair and faeces are not elevated in reindeer infected with a gastrointestinal nematode (*O. gruehneri*) and, therefore, GC levels would not be a good proxy (or bio-indicator) for infection with this parasite (and perhaps similar genera), at least at these low to moderate infection intensities. Our results even raise the question of whether low to moderate infection intensities with helminths are beneficial for the host, a scarcely explored hypothesis in wildlife. Glucocorticoids may still reflect exposure to other environmental stressors in *Rangifer*, such as anthropogenic disturbance (Wasser *et al*., 2011), and provide conservation-relevant information. However, we caution against the use of stress measurements in isolation from other health and population viability indices until we have a better understanding of what the relationships between these measurements are. Although the present study focuses on one species, our results and interpretations are more broadly relevant because gastrointestinal nematodes are ubiquitous in most wildlife populations. Finally, the finding that faecal corticosterone levels may be significantly elevated when faecal cortisol levels are not also underscores the value of quantifying both these steroids. Measuring more than one corticosteroid may provide increased sensitivity to changes in hormone levels and reveal important insights relevant to physiological functioning and conservation efforts.
